# Pneumonia-related pneumatoceles in infants: CT assessment and image-guided treatment

**DOI:** 10.1259/bjrcr.20210191

**Published:** 2021-12-17

**Authors:** Elisa Mercanzin, Pietro Andrea Bonaffini, Antonino Barletta, Francesco Stanco, Clarissa Valle, Paolo Marra, Sandro Sironi

**Affiliations:** 1 Department of Radiology, Papa Giovanni XXIII Hospital, Bergamo, Italy; 2 School of Medicine University Milano Bicocca, Milan, Italy; 3 Department of Neuroradiology, Papa Giovanni XXIII Hospital, Bergamo, Italy

## Abstract

Lung pneumatoceles represent a potential life-threatening complication of pneumonia in infants, especially when they do not spontaneously reabsorb. In this category of patients, scientific literature lacks and no consensus guidelines for management have been proposed. Imaging plays a key role in the diagnosis, characterization, and follow-up of pneumatoceles. Image-guided percutaneous drainage can be considered a safe and effective treatment in children, although it is not widely recognized in newborns and infants. The aim of this case series review is to describe the main CT features of complicated or persistent pneumatoceles in infants and to highlight the potential role of image-guided percutaneous drainage as an effective approach for their treatment. Successful management of four infants affected by pneumonia-related pneumatoceles with percutaneous drainage is presented.

## Introduction

Pneumatoceles are thin-walled gas-filled spaces in the lung, most frequently caused by acute pneumonia, trauma, or aspiration of hydrocarbon fluid. There are limited data on the incidence of pneumatoceles.^1^ However, they are uncommon, thanks to the advent of exogenous surfactant therapy and gentle ventilation strategies, with consequent reduction of ventilator-induced lesions.^2^ The pathogenesis of pneumatoceles is still unclear, although several hypotheses were advanced. According to one of the most accepted theories, lung pneumatoceles develop as a consequence of lung parenchymal necrosis followed by enlargement secondary to a check-valve bronchiolar obstruction, which is due to either pressure from the adjacent pneumatocele or intraluminal inflammatory exudate.^3^

In children, pneumatoceles mainly result as a complication of bacterial pneumonia caused by *Staphylococcus aureus*, more rarely by other Gram+ and Gram- pathogens.^2^ *Staphylococcus aureus* typically causes a bronchopneumonia with singular or multifocal consolidations; abscess formation with cavitation is frequent.^4^

Pneumatoceles tend to spontaneously reabsorb in few weeks–months, but their persistence can lead to severe complications such as cardiorespiratory compromise, tension pneumatocele, pneumothorax and superinfection, with increased risk of mortality.^5^

Imaging is an essential support for physicians in work-up and management of post-pneumonic pneumatoceles. On plain chest radiography (CXR) and CT scan, a pneumatocele manifests as an almost round, thin-walled airspace with adjacent consolidations or ground-glass opacities. These findings, coupled with clinical data, help in the differential diagnosis with other lung diseases such as cysts, cavities, bullae, emphysema and cystic bronchiectasis.^6^ Imaging also plays a critical role in the follow-up, to monitor lesion size and detect complications. In addition, it is helpful to assess the evolution of the underlying pneumonia.

Even if a consensus management algorithm does not exist and few small reports are found in the literature, complicated or persistent symptomatic pneumatoceles can be treated with image-guided percutaneous aspiration and drainage, although some cases require surgery for a complete toilette.^2,7–10^ To support this interventional strategy, we retrospectively reviewed the management of four infants affected by persistent or complicated post-pneumonic pneumatocele, who were successfully treated with image-guided percutaneous drainage.

## Study protocol

The study included infants who were admitted to a single center between November 2013 and December 2019, who received the diagnosis of post-pneumonic pneumatocele. The inclusion criteria were: (a) age < 1-year-old at diagnosis (b) CXR and pre-operative CT evaluation (c) pneumatocele’s treatment with image-guided catheter drainage (IGCD) (either with fluoroscopic, CT or ultrasound guidance) (d) post-operative assessment with CXR or CT and clinical data. The recorded data included the following: age, sex, medical comorbidities, symptoms, and clinical course. CT was used in the pre-operative assessment of pneumatoceles features such as site, dimensions, number, presence of septa, fluid or solid content, wall thickness and/or communications with bronchial tree. Chest CT scans from the apex to the lung bases were acquired with infants lying in the supine position under sedation with intravenous Propofol, with a 64-slice scanner (Brilliance 64-slice; Philips Medical Systems, Best, Netherlands). CT acquisitions parameters were the following: tube voltage, 80 kV; automatic exposure control for tube current; slice thickness, 2.5 mm; images retroreconstruction, 1.00 mm. A single-phase contrast-enhanced CT was performed in one case, with scan acquired in portal venous phase after intravenous contrast injection (Iomeron 350; 26 ml). In the other three cases, no intravenous contrast media was administered.

Percutaneous drainage procedures were performed under general anesthesia: premedication with midazolam (0.5 mg/kg orally) was given in the ward; intubation was performed by dedicated pediatric anesthetists in the angiographic suite after induction with propofol 1–2 mg/kg, fentanyl (0.5–1 mcg/Kg) and rocuronium bromide (1 mg/kg); anesthesia was maintained with sevoflurane under ECG, blood pressure, pulse oximetry, temperature and capnometry monitoring.

Ultrasound-guidance was used for the choice of the entry site that was locally anesthetized with 0.25% bupivacaine (1 mL/kg). Fluoroscopic guidance was used for the pigtail catheter positioning with the Seldinger technique: after puncture of the pneumatocele with a 18G Chiba needle (Chibell, Biopsybell, Mirandola, Italy) the drainage catheter was advance on an Amplatz stiff guidewire (Cook Incorporated, Bloomington, IN). In three patients, 8-Fr catheters (Skater, Argon Medical, Frisco, Texas) were inserted; in one patient a 6-Fr catheter (Skater, Argon Medical, Frisco, Texas) was used. In three cases, a post-procedural CT scan was performed to check catheter position.

After catheter placement, aspiration was performed to collect material for microbiological analyses. Then the catheter was connected to an underwater-seal drainage system, to achieve negative pressure.

Broad-spectrum intravenous antibiotics (cefazolin, 25 mg/kg) were used prophylactically and continued until antibiogram, then targeted. Serial CXRs were performed every 48–72 h until lesion disappearance in all patients; CT was performed in one case during follow-up. To determine the timing of catheter removal, two standard criteria were used: first, >80% pneumatocele reduction confirmed by CXR/CT evaluation; second, no recurrence of air collection on CXR after clamping the catheter for 24 h. After catheter removal, control CXRs were repeated at 24 and 48 h.

## Representative cases

### Case I

**Figure 1.**
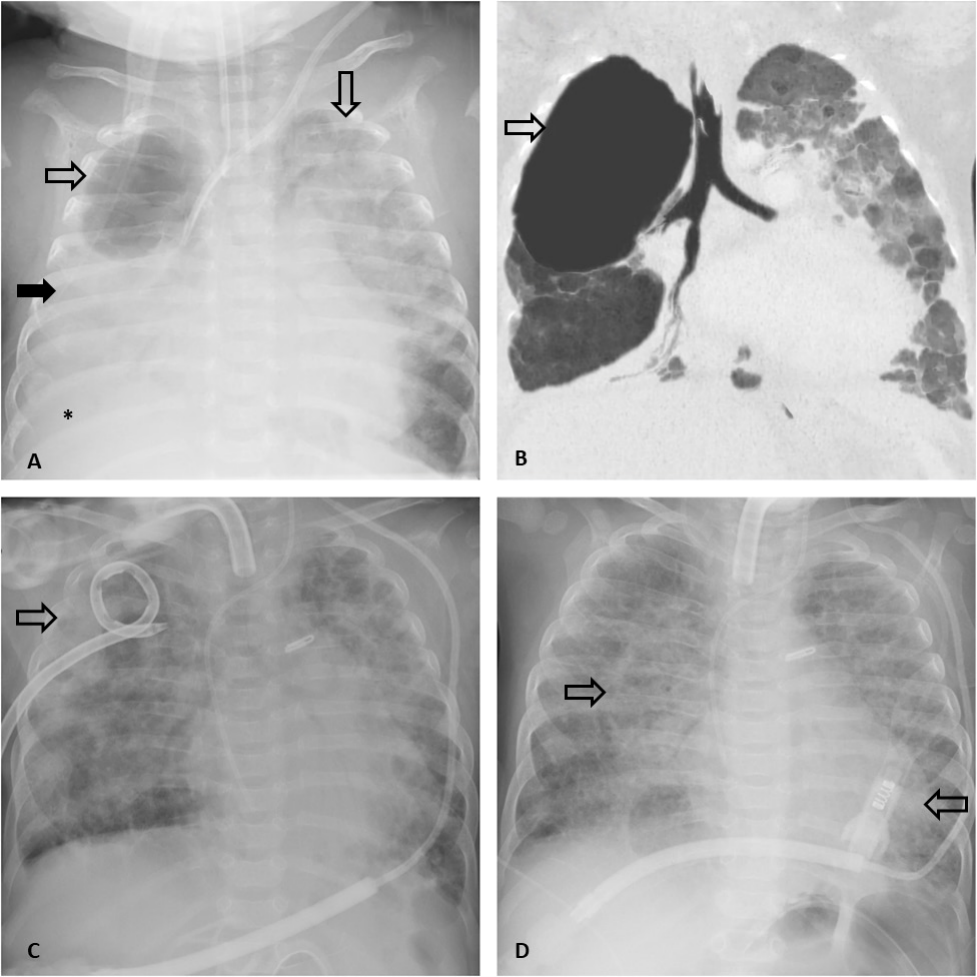
Images from case I. (A) Frontal CXR shows diffuse bilateral consolidations (full black arrow) and pleural effusion (*). In the right upper lobe, a large hypoattenuating area, then confirmed on CT as pneumatocele (not shown), is evident (empty black arrow). (B) MinIP coronal non-contrast chest CT, performed at 10 months of age, shows increase in size of the pneumatocele, appearing as a single thin-walled cavity (septa non-shown) (empty black arrow). (C) CXR demonstrates a pig-tail catheter *in situ* (empty black arrow), with significant decreased size of the pneumatocele. (D) CXR, performed the day after catheter removal shows the complete resolution of the pneumatocele. Persistent bilateral consolidations are still present (empty black arrows) but improved compared to baseline CXR. MinIP, Minimum Intensity Projection.

An extremely preterm low-birth girl (26 weeks gestational age, 473 gr) underwent mechanical ventilation for a neonatal respiratory distress syndrome (RDS). The neonatal period was characterized by several infectious events (coagulase-negative staphylococci, methicillin-resistance *Staphylococcus aureus* (MRSA), *S. epidermidis*). A chest X-ray (CXR) showed bronchopulmonary dysplasia with bilateral consolidations and a hypoattenuating area with a visible thin wall in the right upper lobe (Figure 1a). Due to the progressive increase over time of this hypoattenuating area at serial CXRs, the need for mechanical ventilation and worsening of the cardiorespiratory impairment, the patient underwent CT. A 21 × 19 × 17 mm multiseptated pneumatocele with thin wall in the apical segment of the right upper lung lobe and severe bronchopulmonary dysplasia were confirmed. However, the clinical conditions stabilized with medical treatment. Follow-up CXRs demonstrated further size increase of the pneumatocele. At 10 months of age, a probable *ab ingestis* episode caused an acute worsening of respiratory function, that required a new CT scan. The pneumatocele had increase to 48 × 39 × 54 mm (Figure 1b). Upon shared indication with clinicians, IGCD of the pneumatocele was performed. After the procedure, the medium and superior right lobes recruitment improved and the follow-up CXRs documented the progressive size reduction of the pneumatocele (Figure 1c). Clinical conditions consequently improved, and the drainage could be removed after 26 days (Figure 1d). Unfortunately, the patient died at the age of 1 year and 1 month for a sudden multiorgan failure (MOF) due to cardiac comorbidity.

### Case II

A full-term new-born by cesarean delivery with congenital cardiac defect (Truncus Arteriosus type II) was operated in his first month of life. The post-operative course was complicated by hemodynamic instability with hypotension, sepsis and hypoxia requiring mechanical support. Parenchymal lung consolidation with cavities was detected on CXR and confirmed by CT. *Pseudomonas aeruginosa* was isolated from bronchial aspiration.

During radiological follow-up, the baby developed a 59 × 22 × 31 mm multiseptated pneumatocele in the superior right lobe. The clinical conditions stabilized with mechanical and pharmacological support. After discharge, the child showed well-being and regular growth for 4 months. A follow-up CT scan showed size reduction (24 × 23 × 21 mm) and no septations in the pneumatocele.

However, at 8 months an episode of pneumatocele superinfection occurred and required antibiotic therapy.

Due to pneumatocele persistence and infection recurrence, to prevent new potential complications, an elective IGCD of the pneumatocele was scheduled in the second year of life. Serial CXRs demonstrated the resolution of the pneumatocele. The drainage was closed within 5 days, then removed.

A small residual opacity in the upper right lobe stably persisted at the follow-up CXR with well-being of the child.

### Case III

**Figure 2.**
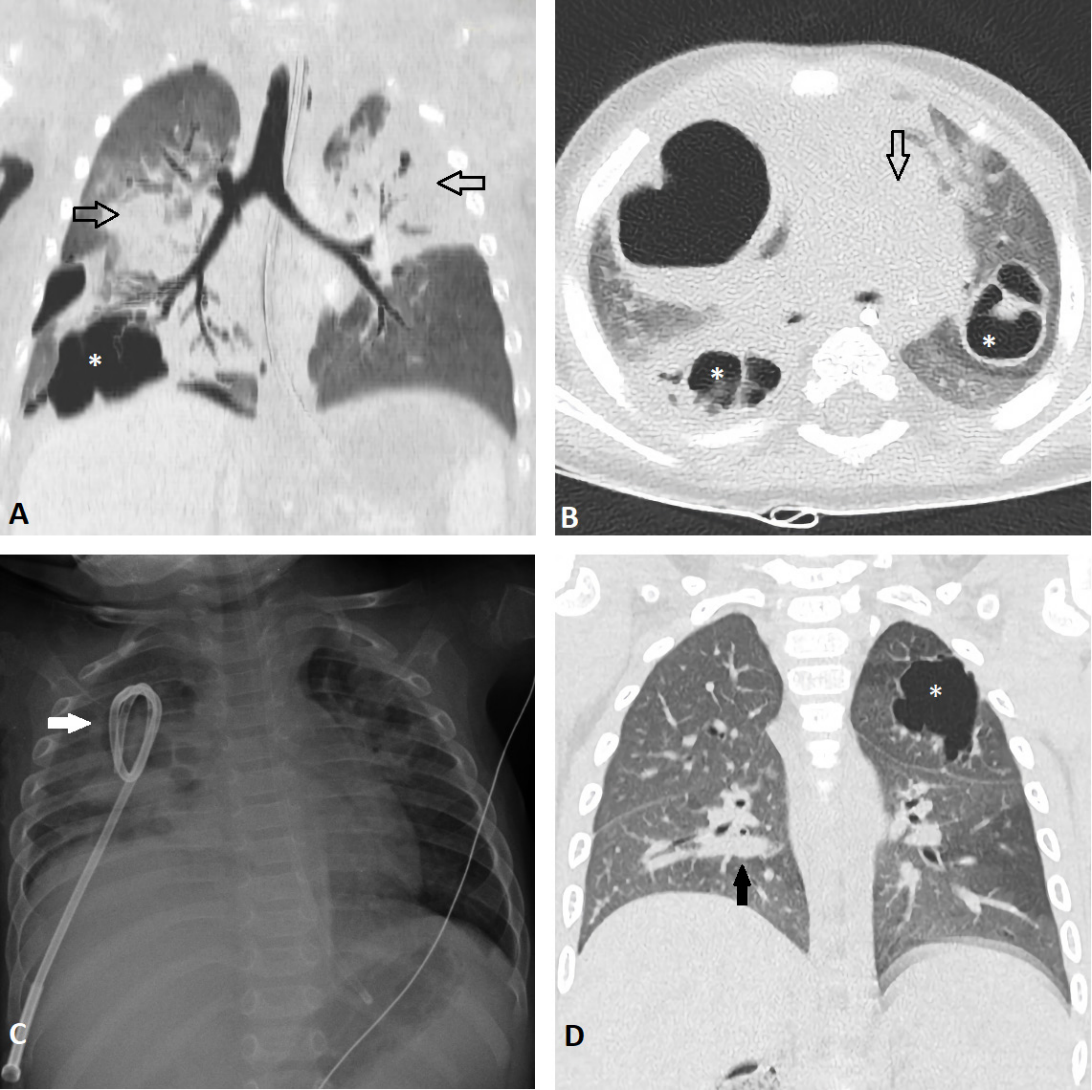
Images from Case III. (A, B) Coronal MinIP and axial CT scans show bilateral consolidations with air bronchogram (empty black arrows), (A) and multiple pneumatoceles, some of which present inner septations (*, A, B). The largest pneumatocele is detected in the upper right lobe. (C) An 8-Fr pig-tail catheter is placed in the largest pneumatocele (full white arrow), under ultrasound and fluoroscopic guidance. (D) Coronal CT scan obtained 5 months later shows resolution of right pneumatoceles and a persistent thin-walled pneumatocele (*) in the upper left lobe. Persistent peribronchial consolidations are also noted in the right lower lobe (full black arrow). MinIP, Minimum Intensity Projection.

A 10-month healthy twin boy, born by eutocic delivery, came to emergency room for dyspnoea. CXR showed bilateral lung consolidations. The laboratory test revealed methicillin-sensitive *Staphylococcus aureus* (MSSA) pneumonia. During the hospitalization in pediatric intensive care unit, there was a sudden worsening of clinical conditions, with development of acute respiratory distress syndrome, septic shock, and MOF. Severe respiratory failure was worsened by onset of barotraumatic pneumothorax, successfully drained. A CT scan showed multiple bilateral consolidations and multiple bilateral pneumatoceles in all lobes, sparing the inferior left lobe (Figure 2a and b). Follow-up CXRs demonstrated a progressive increase in number and size of the pneumatoceles. In combination with antibiotic therapy, IGCD of the largest lesion (46 × 34 × 51 mm) that presented an air fluid-level consistent with abscessualization (Figure 2c), was performed. The drainage was removed after 6 days of clinical improvement. A CT scan performed 5 months later showed resolution of right pneumatoceles; a 30 mm pneumatocele persisted in the contralateral upper lobe (Figure 2d) and the patient remained asymptomatic.

### Case IV

A preterm very low-birth weight girl (24 weeks gestational age, 680 gr), delivered by urgent cesarean section due to placenta previa, required invasive mechanical ventilation until 18 h of life for a neonatal RDS. The infant was successfully treated with surfactant therapy. In the second week of life, an MRSA sepsis occurred, leading to cardiocirculatory and respiratory instability that required invasive and oscillatory ventilation. CXR and CT scan showed a 24 × 20 × 22 mm uniloculated pneumatocele involving the anterior and apical segments of the right superior lobe, associated to diffuse reticular interstitial thickening and parenchymal consolidations suggestive for bronchopulmonary dysplasia. Because of respiratory impairment, IGCD of the pneumatocele was performed; the drainage was successfully removed after 10 days of clinical improvement. The follow-up CXR exams showed stable bronchopulmonary dysplasia without pneumatocele relapse.

## Discussion

Pneumatoceles may represent a severe clinical threaten in infants. Although they spontaneously reabsorb in most cases, they can persist and lead to severe clinical conditions, especially in patients with compromised cardiorespiratory function.

In this study, four infants were affected by bacterial pneumonia during their first year of life. The infections were complicated by the development of pneumatoceles (Table 1).

**Table 1.** Main clinical characteristics of the four cases of pneumatoceles that underwent IGCD procedure

Patient (sex)	Prematurity	RDS	Pathogen involved	Pneumatocele onset (months of life)	Comorbidities
1 (F)	Yes	Yes	MRSA	1	GERD, patent arteriosus duct
2 (M)	No	Yes	P. *aeruginosa*	1	Truncus arteriosus type II
3 (M)	Yes	Yes	MSSA	10	None
4 (F)	Yes	Yes	MRSA	<1	Patent arteriosus duct. NEC. sepsis

F, female; GERD, gastro-esophageal reflux disease;IGCD, image-guided catheter drainage; M, male; MRSA, methicillin-resistant Staphylococcus aureus; MSSA, methicillin-sensitive Staphylococcus aureus; NEC, necrotizing enterocolitis; RDS, respiratory distress syndrome.

Three patients were premature and developed postnatal RDS as predisposing factor. In all cases, CXR suggested the presence of pneumatoceles and CT not only confirmed the diagnosis but also characterized the lesions (Table 2). Based on this accurate imaging assessment, we found that pneumatoceles were single and dominant in most cases, septated in the half, and most commonly located in the right upper lobe. Of note, communication with the bronchial tree was present in three cases. CT was also useful to plan the interventional procedure: in case of multiple or multiseptated pneumatoceles, CT guided the most feasible percutaneous route for drainage.

**Table 2.** CT findings of post-pneumonia pneumatoceles

Patient (sex)	Number	Location	Dimensions (cm)	Presence of septa	Communications with the bronchial tree
1 (F)	Single	RUL	5 × 4×4	Yes	Yes
2 (M)	Single	RUL	2.5 × 2×2	Yes	Yes
3 (M)	Multiple	RUL, ML, RIL, LUL	4.5 × 3.1×5.3	No	Yes
4 (F)	Single	RUL	2.4 × 2.2×2.2	No	No

IGCD, image-guided catheter drainage; LUL, left upper lobe; ML, middle lobe; RIL, right inferior lobe; RUL, right upper lobe.

In the general population, conservative pharmacological therapy is the less invasive therapeutic strategy for pneumatoceles but potentially ineffective in complicated cases. Surgery is an option, but it is burdened by higher morbidity, especially in critical patients, children and newborns. Therefore, in selected cases IGCD could be considered an effective and minimally invasive alternative treatment.^2,7–10^

To our knowledge, the only pneumatocele treatment algorithm was proposed for children by Imamoğlu et al.^7^ They recommend IGCD procedures in case of persistent symptoms (>6 months), >50% lung involvement, severe atelectasis, bronchopleural fistulae, thin-walled abscess, and bad tolerance to follow-up. Conversely, they propose follow-up in case of simple pneumatocele in asymptomatic patients with 10–50% lung involvement, with good compliance to follow-up. The algorithm proposes surgical excision in case of complications, such as abscessualization of the cavity or persistence of the pneumatocele with thickened wall for more than 6 months. Of note, the algorithm proposed by Imamoğlu et al^7^ did not consider comorbidities. Our patients differ from those reported by Imamoğlu et al^7^: first, they are newborns and infants; second, their clinical conditions were complicated by prematurity, RDS, congenital heart diseases or NEC. Therefore, consensus guidelines lack for this category of patients. In our experience, we resorted to IGCD due to the worsening of respiratory function in three cases or persistence of the pneumatocele (over 6 months) in one.

The good outcome was proved by radiological assessment with CXRs, which confirmed the complete regression of pneumatoceles and by clinical data, which demonstrated the improvement of respiratory function in all cases. Despite successful treatment, one infant died 3 months after drainage for a sudden MOF unrelated to pneumatocele.

In conclusion, persistent or complicated pneumatoceles are associated with increased morbidity and mortality in infants and no consensus clinical guidelines are available for their management. Our experience suggests a pivotal role of CT in the pre-operative assessment and characterization of pneumatoceles and safety and efficacy of mini-invasive IGCD.

## Learning points

- Pneumatoceles in infants are a possible complication of bacterial pneumonia.
- Large or multiple pneumatoceles can compromise lung function, in particular if there are other comorbidities.
- Chest CT is useful to correctly diagnose pneumatoceles, to define their characteristics in addition to evaluate lung parenchyma.
- After CT, image-guided catheter drainage is a possible and effective treatment in these cases.

## References

[1] Hansell DM, Bankier AA, MacMahon H, McLoud TC, Müller NL, Remy J. Fleischner society: glossary of terms for thoracic imaging. *Radiology* 2008; **246**: 697–722. doi: 10.1148/radiol.2462070712 18195376

[2] Kumar J, Mukhopadhyay K, Bhatia A. Successful percutaneous drainage of pneumatoceles in an extremely low-birthweight infant. *BMJ Case Rep* 2018; **2018**: bcr-2017-222630. doi: 10.1136/bcr-2017-222630 PMC5786982 29374641

[3] Quigley MJ, Fraser RS. Pulmonary pneumatocele: pathology and pathogenesis. *AJR Am J Roentgenol* 1988; **150**: 1275–77. doi: 10.2214/ajr.150.6.1275 3259364

[4] Vilar J, Domingo ML, Soto C, Cogollos J. Radiology of bacterial pneumonia. *Eur J Radiol* 2004; **51**: 102–13. doi: 10.1016/j.ejrad.2004.03.010 15246516

[5] Maranella E, Conte E, Di Natale C, Coclite E, Di Fabio S, et al. Disseminated, large-sized neonatal pneumatoceles: the wait-and-see strategy. *Pediatr Pulmonol* 2014; **49**: E69-71. doi: 10.1002/ppul.22831 23794463

[6] Lee KC, Kang EY, Yong HS, Kim C, Lee KY, Hwang SH, et al. A stepwise diagnostic approach to cystic lung diseases for radiologists. *Korean J Radiol* 2019; **20**: 1368–80. doi: 10.3348/kjr.2019.0057 31464115 PMC6715565

[7] Imamoğlu M, Cay A, Koşucu P, Ozdemir O, Cobanoğlu U, Orhan F, et al. Pneumatoceles in postpneumonic empyema: an algorithmic approach. *J Pediatr Surg* 2005; **40**: 1111–17. doi: 10.1016/j.jpedsurg.2005.03.048 16034754

[8] DiBardino DJ, Espada R, Seu P, Goss JA. Management of complicated pneumatocele. *J Thorac Cardiovasc Surg* 2003; **126**: 859–61. doi: 10.1016/s0022-5223(03)00367-2 14502169

[9] Zuhdi MK, Spear RM, Worthen HM, Peterson BM. Percutaneous catheter drainage of tension pneumatocele, secondarily infected pneumatocele, and lung abscess in children. *Crit Care Med* 1996; **24**: 330–33. doi: 10.1097/00003246-199602000-00024 8605809

[10] Kaplan LJ, Trooskin SZ, Santora TA, Weiss JP. Percutaneous drainage of recurrent pneumothoraces and pneumatoceles. *J Trauma* 1996; **41**: 1069–72. doi: 10.1097/00005373-199612000-00026 8970569

